# A combined targeted mutation analysis of *IRF6* gene would be useful in the first screening of oral facial clefts

**DOI:** 10.1186/1471-2350-14-37

**Published:** 2013-03-20

**Authors:** Yah-Huei Wu-Chou, Lun-Jou Lo, Kuo-Ting Philip Chen, Chun-Shin Frank Chang, Yu-Ray Chen

**Affiliations:** 1 Department of Medical Research, Chang Gung Memorial Hospital, No. 5, Fushing Street, Kweishan, Taoyuan, Taiwan; 2 Craniofacial Research Center, Chang Gung Memorial Hospital, Linkou, Taiwan; 3 Graduate Institute of Clinical Medical Science, Chang Gung University, Taoyuan, Taiwan; 4 Department of Plastic and Reconstructive Surgery, Chang Gung Memorial Hospital, Linkou, Taiwan; 5 Graduate Institute of Dental and Craniofacial Science, Chang Gung University, Taoyuan, Taiwan

**Keywords:** *IRF6* gene, Mutation analyses, Orofacial clefts

## Abstract

**Background:**

Interferon Regulatory Factor 6 (*IRF6*) is a member of the IRF family of transcription factors. It has been suggested to be an important contributor to orofacial development since mutations of the *IRF6* gene has been found in Van der Woude (VWS) and popliteal pterygium syndromes (PPS), two disorders that can present with isolated cleft lip and palate. The association between *IRF6* gene and cleft lip and palate has also been independently replicated in many populations.

**Methods:**

We screened a total of 155 Taiwanese patients with cleft lip with or without cleft palate (CL/P); 31 syndromic (including 19 VWS families), 44 non-syndromic families with at least two affected members, and 80 non-syndromic patients through a combined targeted, polymerase chain reaction (PCR)-based mutation analysis for the entire coding regions of *IRF6* gene.

**Results:**

We found 11 mutations in 57.89% (11/19) of the VWS patients and no *IRF6* mutation in 44 of the non-syndromic multiplex families and 80 non-syndromic oral cleft patients. In this *IRF6* gene screening, five of these mutations (c.290 A>G, p.Tyr97Cys; c.360-375 16 bp deletion, p.Gln120HisfsX24; c.411_412 insA, p.Glu136fsX3; c.871 A>C, p.Thr291Pro; c.969 G>A, and p.Trp323X) have not been reported in the literature previously. Exon deletion was not detected in this series of *IRF6* gene screening.

**Conclusions:**

Our results confirm the crucial role of *IRF6* in the VWS patients and further work is needed to explore for its function in the non-syndromic oral cleft with vary clinical features.

## Background

Orofacial clefts are common congenital malformations which require long-term treatment and patient care. They are usually classified as either cleft lip with or without cleft palate (CL/P) or cleft palate only (CP), based on differences in embryological development [1,2]. Approximately 70% of cases of orofacial clefting occur as isolated entities with no other apparent cognitive or craniofacial structural abnormalities and are commonly termed ‘isolated, non-syndromic CL/P (NSCL/P)’; while many syndromic forms have simple Mendelian patterns of inheritance and therefore are amenable to disease gene identification [3-5]. Previous genetic and epidemiological studies have indicated that the causes of nonsyndromic CL/P are multifactorial, with both genetic and environmental factors contributing to the phenotype [6-9].

Of the large number of candidate genes thought to contribute to orofacial clefting, Interferon Regulatory Factor 6 (*IRF6*) is the only gene that has shown a convincing degree of consistency across studies [10,11]. Mutations in *IRF6* were first identified in the autosomal dominant van der Woude syndrome (VWS; OMIM 119300), which is traditionally recognized as a monogenic syndrome characterized by lip pits, clefting of the primary or secondary palate, and hypodontia [12]. Van der Woude syndrome (VWS) is estimated as most common syndromic form of oral clefts, accounting for 2% of all cases of cleft lip and palate, and has the most closely similarities in phenotype with that of common non-syndromic forms [13-15]. Up to date, at least 200 different mutations in the *IRF6* gene have been described with the majority being protein truncation mutations (nonsense and frameshifts) or missense mutations [16]. Given the overlapping phenotype of VWS with isolated CL/P, in searching the gene for mutations, non-pathogenic variants in *IRF6* gene have also been found to be significantly associated with nonsyndromic oral clefting in many different populations and ethnic groups [10,17-23].

In order to determine the contribution of mutations in *IRF6* to oral clefts in the Taiwanese population, we applied a combination of targeted, PCR-based experimental approaches (including polymerase chain reaction (PCR), Denature High Performance Liquid Chromatography (DHPLC), Multiplex ligation-dependent probe amplification (MLPA), TOPO cloning and DNA sequence analysis) for a cohort of oral cleft patients from Craniofacial center of Chang Gung Memorial Hospital.

## Methods

### Study subjects

All patients were clinically assessed by the plastic surgeon and diagnosis as CL/P or CP based on clinical examination, medical records, and a detailed questionnaire through Chang Gung Craniofacial Center. A total of 155 patients with CL/P were included (31 syndromic, 44 non-syndromic families with at least two affected members, and 80 non-syndromic patients). Diagnostic criteria for individuals to be considered affected with VWS included CLP or CPO, and at least one affected individual in the family with an anomaly in the lower lip, generally bilateral pits. Cases in this series (collected from 1997-2011) were selected only from cleft patients, thus VWS patients presenting only with lower lip pits were not included. In addition to these families, a total of 100 healthy volunteers with no family history of VWS and cleft lip and/or cleft palate were recruited as a control group for genetic analysis. This study was approved by Chang Gung Medical Foundation Institutional Review Board (No. 98-2610C) and written informed consent was obtained from all patients or their parents prior to participation in this study.

### Molecular genetics analysis

#### DNA extraction and PCR amplification

Genomic DNA from blood were prepared from all the study subjects by standard proteinase K digestion and phenol/chloroform extraction using PUREGENE DNA purification kit from GENTRA (Minneapolis, Minn, USA).

The exon-intron structure and sequence for entire *IRF6* gene were assessed from the National Center for Biotechnology Information (NCBI) web site and published literature. The oligonucleotide primers for amplification of each exon and adjacent splice sites and at least 40 bp upstream of the acceptor splice sites and least 40 bp downstream of donor sites were designed with Primer3 (http://frodo.wi.mit.edu/primer3/input.htm). PCR amplification was performed using an automated thermocycler 9600/9700 (Applied Biosystem) in a final volume of 25 ul containing 25 ng of template DNA, 2.5 ul 10× reaction buffer(Sigma), 0.8 ul 25 mM of dNTP, 5 ng of each of the primers and 0.25 units of Taq polymerase. The amplification conditions were adjusted to the best condition for each exon and were: denaturation at 94°C for 2 minutes; 30 cycles each at 94°C for 30 seconds, 56°C or 60°C for 30 seconds, and 72°C for 30 seconds; and 72°C for 5 minutes.

#### Mutation detection using denature high performance liquid chromatography

PCR products were denatured and reannealed prior to DHPLC analysis using a PCR program of briefly heating to 95°C for 5 min followed by slow cooling to 25°C at a rate of 1°C every 30s. The products were then run on a WAVE-3500 Transgenomic WAVE™ machine equipped with a DNASep column and WAVE Optimized™ Buffer (Transgenomic Inc., Crewe, UK). The oven temperatures for optimal heteroduplex separation were resolved with Navigator v1.5.2 software, which gives a computer-assisted determination of melting profile and analytical conditions for each fragment. Wild-type DHPLC elution profiles were characterized for each exon fragment by analyzing at least two normal control DNA samples.

#### Dissection of genomic rearrangement for candidate genes using MLPA

Multiplex ligation-dependent probe amplification (MLPA) was performed with 100 ng of genomic DNA according to manufacturer’s instructions using the SALSA MLPA KIT P304-A1 IRF6 (MRC-Holland, Amsterdam, the Netherlands). This MLPA assay was designed to detect deletions/duplications of one or more exons of the *IRF6* gene. Probe amplification products were run on an ABI 3730 DNA Analyzer using GS500 size standard (Applied Biosystems). MLPA peak plots were visualized using Genemapper Software version 3.7 (Applied Biosystems). Non-normalized values for peak height and peak area were then exported from Genemapper Software version 3.7 to an Excel template. Normalization of data and calculation of dosage ratios were performed as described at http://www.mlpa.com.

#### TOPO TA cloning and DNA sequencing

We performed TOPO TA cloning to distinguish allelic status of PCR products with aberrant DNA sequence. This strategy allows PCR inserts to ligate efficiently with the topoisomerase I-bound vector (Invitrogen, CA, USA). At least 10 colonies were selected and analyzed by restriction enzyme-EcoRI to confirm the presence of the insert. Finally, plasmids were further confirmed by sequencing using ABI prism 3730 automated DNA Sequencer (Applied Biosystems). Sequence was analyzed using Sequencing Analysis 5.2 software. Autoassembler computer program (Applied Biosystems) was used for sequence alignments and analysis. DNA sequence variants were confirmed by sequencing the opposite strand of the PCR product. Mutations and polymorphisms were sequenced in 96 control chromosomes from ethnically matched, unaffected individuals.

## Results

We screened a total of 155 patients with CL/P; 31 syndromic, 44 non-syndromic families with at least two affected members, and 80 non-syndromic patients through a procedure of mutation analysis for the entire PCR-amplified protein coding regions of *IRF6.* Eleven different mutations occurring in exons 3, 4, 5, and 7 of *IRF6* gene were identified in the VWS patients (11/19, 57.89%). (Table 1) None was detected in 44 of the non-syndromic multiplex families and 80 non-syndromic oral cleft patients. Seven mutations (p.Ala16Val, p.Trp28X, p.Arg84Cys, p.Arg84His, p.Lys89Glu, p.Tyr97Cys, and p.Gln120HisfsX24) affected the DNA-binding domain, which is involved in DNA interactions. Three mutations (p.Thr291Pro, p.Trp323X, and p.Cys347Phe) were found in the Smad-interferon regulatory factor-binding domain that is critical for both homo- and hetero-dimerization of IRF6 protein. There were one mutations (p.Lys137fsX3) detected downstream of the DNA-binding domain. In the present study, all affected members were heterozygous for their respective mutation and five of these mutations (p.Tyr97Cys, p.Gln120HisfsX24, p.Glu136fsX3, p.Thr291Pro, and p.Trp323X) have not been reported in the literature previously (Additional file 1: Figure S1). We also apply the multiplex ligation-dependent probe amplification technique (MLPA) to test for single/multiple exon deletions/duplication within the *IRF6* gene. However, there were no such mutations detected in this study. For those multiplex families, mutations detected in VWS-1, VWS -6, VWS -N9, and VWS-N90 are all cosegregated with their affected members in the family (data not shown).

**Table 1:** ***IRF6 *mutations identified in VWS patients**

**Pt no.**	**SEX**	**Family history**	**Cleft type**	**Exon**	**IRF6 mutation**	**aa changed**	**Lip pit/nodules**
VWS-1	M	positive	BCLP	7	c. 1040 G>T	p. Cys347Phe	2 pits
VWS-2	F	negative	BCLP	4	c. 265 A>G	p. Lys89Glu	2 pits
VWS-3	F	negative	RCLP	--	--	--	2 asymmetric pits
VWS-4	F	negative	BCLP	5	c. 411_412 insA	p. Lys137fsX3	2 pits
VWS-5	M	negative	BCL	3 + 4	c. 47 C>T, c. 290 A>G	p.Ala16Val, p.Tyr97Cys	2 pits
VWS-6	M	positive	BCLP	7	c. 969 G>A	p. Trp323X	2 pits
VWS-7	M	negative	RCLP	--	--	--	2 pits
VWS-8	F	negative	BCLP	4	c. 250 C>T	p. Arg84Cys	2 pits
VWS-9	F	negative	BCLP	4	c. 333 T>C	p. Tyr111Tyr	2 pits
VWS-N9	M	positive	BCLP, incomplete	4	c. 251 G>A	p. Arg84His	2 pits
VWS-11	F	negative	BCLP	4	c. 251 G>A	p. Arg84His	2 pits
VWS-12	M	negative	BCLP	--	--	--	2 pits
VWS-N90	F	positive	BCLP	4	c. 360-375 16 bp del	p. Gln120HisfsX24	2 pits
VWS-14	F	negative	LCL	--	--	--	2 asymmetric pits
VWS-15	M	negative	BCLP	--	--	--	2 pits
VWS-N93	M	negative	LCLP	3	c. 83 G>A	p. Trp28X	2 pits
VWS-N98	F	positive	LCLP	--	--	--	2 asymmetric pits
old pt	M	positive	LCLP	5	c.411 G>A	p. Lys137Lys	2 pits
194	M	negative	BCLP	7	c.871 A>C	p. Thr291Pro	2 pits

## Discussion

We have identified 11 different *IRF6* mutations in VWS patients and none in 44 of the non-syndromic multiplex families and 80 non-syndromic oral cleft patients. In the present study, all affected members were heterozygous for their respective mutation and five of these mutations (p.Tyr97Cys, p.Gln120HisfsX24, p.Glu136fsX3, p.Thr291Pro, and p.Trp323X) have not been reported in the literature previously.

Interferon Regulatory Factor 6 (*IRF6*) is a member of transcription factors that contain a highly conserved helix-turn-helix DNA-binding domain and a less conserved SMAD-IRF-binding domain [12]. It has been suggested to be important contributor to orofacial development since mutations of the *IRF6* gene have been found in Van der Woude (VWS) and popliteal pterygium syndromes (PPS), two disorders that can clinically resemble an isolated cleft lip and palate [12]. They were detected in approximately 70% of VWS and 97% of PPS cases [16]. Microdeletion of 1q32–q41 is relatively rare, and only a few cases have been reported in the medical literature [24-26]. While the majority of the VWS mutations were spread over exons 3, 4, 7, 8 and 9, the majority of PPS mutations were concentrated in exons 3 and 4 [16,27]. Basically, our results support a non-random distribution of *IRF6* mutations in VWS. More recently, patients with nonsyndromic oral clefts have also been reported to have *IRF6* mutation [27-30]. Two authors have observed *IRF6* mutations among families with mixed cleft phenotype, but they were unable to exclude VWS completely [28,29]. Therefore, molecular differentiation between these overlapping cleft syndromes is absolutely essential before counseling can be given to family members.

The incidence of VWS among cleft patients in our population is 0.73 to 0.98 per 100,000, which is lower than those reported from other studies [31]. The result of *IRF6* mutation screening for our VWS is also low (57.89%). All these VWS cases were selected from cleft patients, thus VWS patients presenting only with lower lip pits were not included. The patients in our series had more severe types of cleft, with a higher incidence of bilateral complete cleft lip and palate than given in other reports. The size, shape, and depth of the pits varied among patients beyond oral cleft phenotypes. The associated anomalies found in our series included hemangioma, hypertelorism, syngnathism, protruding ears, ventricular septal defect, toe and foot anomaly, and undescended testis. All the differences in the distribution of the different types of cleft, the penetrance, and familial occurrence between our study and other reports may be explained by ethnic and/or nongenetic environmental factors.

Lip and palate formations are the consequence of a complex processes that involves cell proliferation, cell differentiation, cell adhesion, and apoptosis. In theory, failure anywhere in these processes can lead to clefts [2,32]. We hypothesize that *IRF6* may play different roles in combination with other genetic and/or environmental factors during the embryonic development [33,34]. Information regarding the profile of gene-gene or gene-environment interaction or network for the craniofacial development should also be considered. Meta analysis of mutation-proved *IRF6*-related phenotypes might provide another useful indicator for the diagnosis of orofacial cleft in the near future.

## Conclusions

This is one of the largest single reports of *IRF6* mutation screens applying a combined targeted mutation analysis. Our results confirm the crucial role of *IRF6* in the VWS patients and further work is needed to explore for its function in the non-syndromic oral cleft with vary clinical features.

## Abbreviations

CL/P: Cleft lip with or without cleft palate; CP: Cleft palate only; NSCL/P: Non-syndromic CL/P; VWS: Van der Woude syndrome; PPS: Popliteal pterygium syndromes; PCR: Polymerase chain reaction; DHPLC: Denature high performance liquid chromatography; MLPA: Multiplex ligation-dependent probe amplification.

## Competing interests

The authors declare that they have no competing interests.

## Authors’ contributions

YHWC conceived and designed the study. The study subjects were assessed by plastic surgeons LJL, KTC, CSC, and YRC at Chang Gung Craniofacial Center. YHWC performed all the lab data analysis, interpreted the results and drafted the manuscript. All authors provide expertise and approved the final manuscript.

## Pre-publication history

The pre-publication history for this paper can be accessed here:

http://www.biomedcentral.com/1471-2350/14/37/prepub

## Supplementary Material

Additional file 1: Figure S1 The chromatograms of novel mutations in VWS patients. Click here for file
