# Supplementary material for: A combined targeted mutation analysis of IRF6 gene would be useful in the first screening of oral facial clefts
Source: BMC Med Genet. 2013 Mar 20;14:37. doi: 10.1186/1471-2350-14-37 (PMC3606492; doi:10.1186/1471-2350-14-37)

Supplementary Figure 1

The chromatograms of novel mutations in VWS patients

VWS-5 Exon 4 c.290 A>G, p.Tyr97Cys

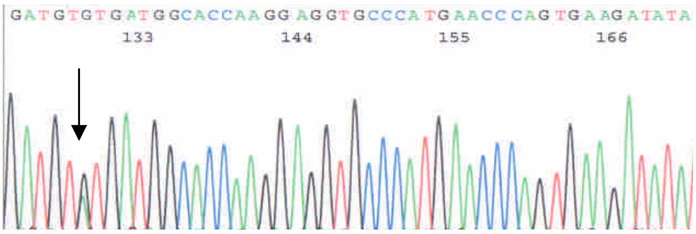

VWS-N90 Exon 4 c.360-375 16bp deletion, p.Gln120HisfsX24

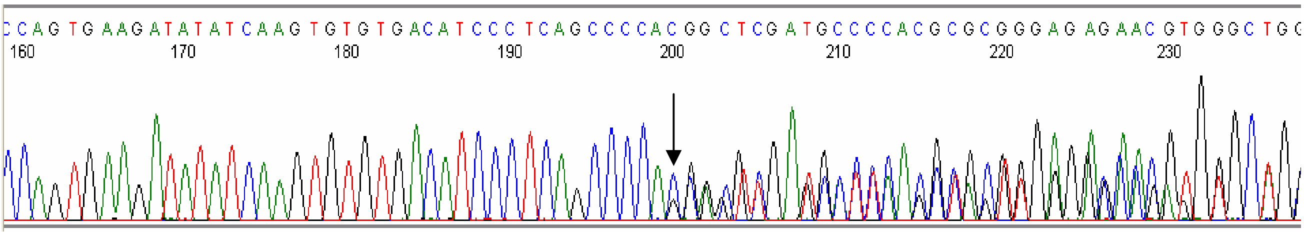

VWS-4 Exon 5 c. 411_412 insA, p.Glu136fsX3

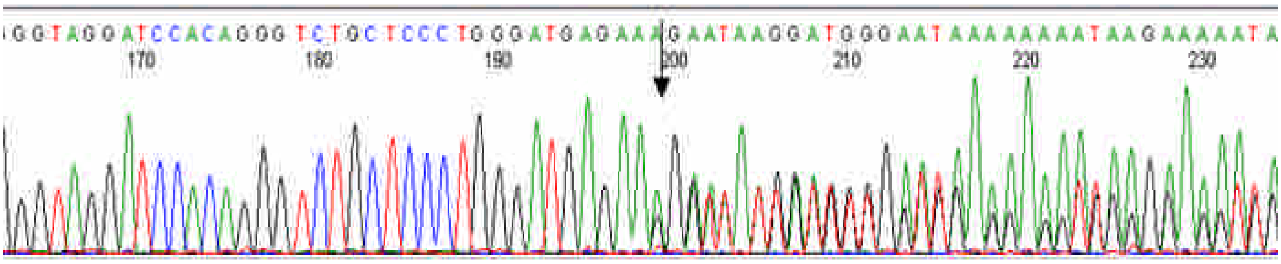

194Pan Exon 7 c.871 A>C, p.Thr291Pro

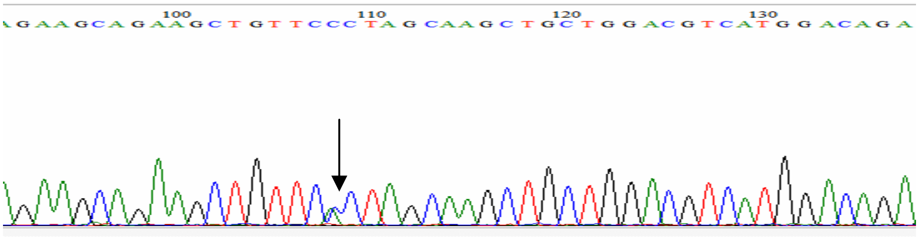

VWS-6 Exon 7 c.969 G>A, p.Trp323X

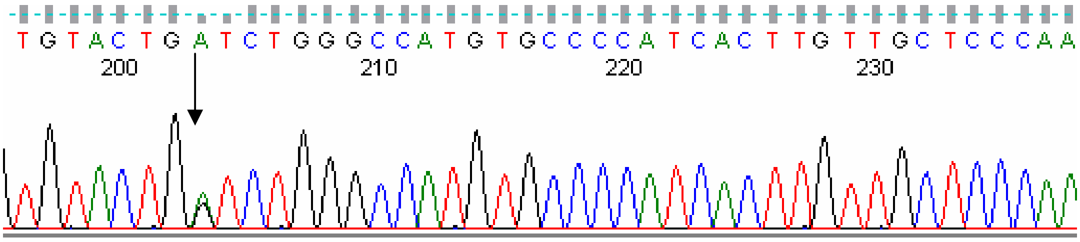

Supplement: Additional file 1: Figure S1 — The chromatograms of novel mutations in VWS patients. [file 1471-2350-14-37-S1.pdf]
